# Packages of Care for Schizophrenia in Low- and Middle-Income Countries

**DOI:** 10.1371/journal.pmed.1000165

**Published:** 2009-10-20

**Authors:** Jair de Jesus Mari, Denise Razzouk, Rangaswamy Thara, Julian Eaton, Graham Thornicroft

**Affiliations:** 1 Department of Psychiatry, Universidade Federal de São Paulo, São Paulo, Brazil; 2 Health Services and Population Research Department, Institute of Psychiatry, King's College London, United Kingdom; 3 Schizophrenia Research Foundation, Chennai, India; 4 CBM International, West Africa Region, Abuja, Nigeria; London School of Hygiene and Tropical Medicine, United Kingdom

## Abstract

In the third in a series of six articles on packages of care for mental disorders in low- and middle-income countries, Jair Mari and colleagues discuss the treatment of schizophrenia.

*This is the third in a series of articles highlighting the delivery of “packages of care” for mental health disorders in low- and middle-income countries. Packages of care are combinations of treatments aimed at improving the recognition and management of conditions to achieve optimal outcomes.*

**Summary Points**

- It is estimated that about 41.7 million people need treatment for schizophrenia and related disorders in low- and middle-income countries (LMICs). The majority of these cases are concentrated in Asia (70%) and Africa (16%).
- In countries with low resources, general physicians and primary health care workers can be trained to recognise and treat people with psychotic disorders in the community. Health systems can scale up such interventions across all routine-care settings by training general physicians and primary health care workers to recognise and treat clients with schizophrenia with effective, evidence-based interventions.
- First- and second-generation antipsychotics (FGAs and SGAs) are similarly effective in the acute treatment of psychotic symptoms. In addition, a number of trials have shown the efficacy of psycho-educational strategies to improve adherence to antipsychotics, to decrease relapse and readmission rates, and to have a positive impact in social functioning of family members and patients.
- A package of care combining low doses of conventional antipsychotics along with brief and simple psycho-educational interventions is recommended as an important strategy to decrease the treatment gap for schizophrenia in LMICs.
- The combination of FGAs and psycho-educational interventions are more cost-effective than the use of drugs alone.

## Introduction

The schizophrenia syndrome, a relatively rare mental disorder, comprises thought disorder, unusual beliefs (delusions), misperceptions (mainly auditory hallucinations), cognitive and affective symptoms, and negative symptoms such as blunted affect, lack of motivation, and an experience of emptiness. The first episode of schizophrenia, which usually occurs when patients are in their early twenties, may have an abrupt or insidious onset. Introspection, defensiveness, and eccentricity can be part of the premorbid personality. Before the onset of psychotic symptoms, social performance and interpersonal relationships can be altered or near to normal. Box 1 shows the International Classification of Disease (ICD) 10 diagnostic criteria for schizophrenia [1].

**Box 1. International Classification of Diseases 10 Criteria for Schizophrenia**

One of symptoms (a–d), or two of symptoms (e–i) should be present for most days in the past month.

- Thought echo, thought insertion or withdrawal, and thought broadcasting;
- Delusions of control, influence, or passivity, clearly referred to body or limb movements or specific thoughts, actions, or sensations; delusional perception;
- Hallucinatory voices giving a running commentary on the patient's behaviour, or discussing the patient among themselves, or other types of hallucinatory voices coming from some part of the body;
- Persistent delusions of other kinds that are culturally inappropriate and completely impossible, such as religious or political identity, or superhuman powers and abilities (e.g. being able to control the weather, or being in communication with aliens from another world);
- Persistent hallucinations in any modality, when accompanied either by fleeting or half-formed delusions without clear affective content, or by persistent over-valued ideas, or when occurring every day for weeks or months on end;
- Breaks or interpolations in the train of thought, resulting in incoherence or irrelevant speech, or neologisms;
- Catatonic behaviour, such as excitement, posturing, or waxy flexibility, negativism, mutism, and stupor;
- “Negative” symptoms such as marked apathy, paucity of speech, and blunting or incongruity of emotional responses, usually resulting in social withdrawal and lowering of social performance; it must be clear that these are not due to depression or to medication;
- A significant and consistent change in the overall quality of some aspects of personal behaviour, manifest as loss of interest, aimlessness, idleness, a self-absorbed attitude, and social withdrawal.

These symptoms are shared by most major classification systems, with some differences in duration of illness, or boundaries of subtypes of disorders.

Source: World Health Organization 1992 [1].

The incidence and prevalence of schizophrenia vary substantially across sites, with a higher incidence and prevalence among males, migrants, and those living in urban environments [2]–[4]. Worldwide, the median incidence of schizophrenia is around 1.5 per 10,000 inhabitants [2], and the lifetime morbid risk of developing schizophrenia is near 0.7% [3]. The incidence ratio for men to develop schizophrenia relative to women is 1.42 [5].

People with schizophrenia have a 2–3-fold higher mortality rate than the general population, a differential gap that has increased over recent decades [4],[6] and that now amounts to an average loss of 25–30 y of potential life among people with schizophrenia [7],[8]. There are several reasons for this differential mortality gap. Patients with schizophrenia have an increased prevalence of metabolic syndrome and its components, which are risk factors for cardiovascular disease and type 2 diabetes [7]. They also have higher rates of smoking and reduced physical activity than the general population. Furthermore, a 10-y longitudinal study in China showed that both the all-cause mortality rate and the suicide rate were higher among men with schizophrenia than among women with the disorder [9]. In general, 28% of the excess mortality associated with schizophrenia is attributable to suicide and 12% to accidents [10]. Finally, social factors such as poverty and reduced access to medical care and the adverse metabolic side effects associated with psychotropic treatments combine to increase the risk of cardiovascular diseases, metabolic syndrome, and sudden deaths among people with schizophrenia [8].

Although the incidence of schizophrenia is low, its early onset, long duration, and severe disability make it a leading contributor to the burden of disease in developing countries [11]. Many low- and middle-income countries (LMICs) have fewer than one psychiatrist per 100,000 population (Table S1). Throughout most of Africa, Melanesia, and Polynesia there is inadequate personnel to treat mental disorders. In particular, there is a scarcity of psychiatric nurses [12]. This lack of human resources is a major contributor to a worldwide treatment gap for schizophrenia [13],[14]. It is estimated that around 41.7 million people with schizophrenia are in need of care in LMICs (Table S1), a worrying statistic given that a delay in treatment of psychosis in LMICs is highly associated with poor outcomes and increasing levels of disabilities [15],[16].

In this article, we focus on the effective management of schizophrenia in LMICs. We review the available evidence on the efficacy of treatments and delivery of care strategies in LMICs. Because this evidence is limited, we also consider evidence from systematic reviews, meta-analyses, and key trials from high-income countries (HICs). On the basis of our review, we propose a package of care—a combination of treatments designed to improve the recognition and management of conditions to achieve optimal outcomes—for schizophrenia.

## The Evidence on the Treatment of Schizophrenia

### Detection of Schizophrenia

A necessary preliminary stage to assist people with a recent onset of schizophrenia is case finding [17]. Detection at the prodromal stage is at present ethically problematic, for example, in relation to providing medication to the proportion of at-risk cases who will not develop the condition [18]. Nevertheless, in America, Europe, and Australasia, the use of specialized early intervention teams, which aim to prevent or delay the transition to a psychotic disorder by identifying individuals with subthreshold symptoms at high risk of developing psychosis [19],[20], is now well established, although with conflicting results [21]–[23].

Where case finding is directed towards identifying individuals who have already “converted” from the prodromal stage to meet the diagnostic criteria for schizophrenia, there is emerging evidence from HICs that reducing the duration of untreated psychosis can benefit patients. The number of relapses may be reduced [24], the long-term clinical outcome may be improved [25], and suicide rates may fall [26]. Nevertheless, despite a consensus about the potential public-health benefits of the early detection of schizophrenia [27], there is signal lack of evidence for such benefits in LMICs. Furthermore, there is an ongoing debate about whether the long-term course and outcome of schizophrenia is more benign in low-income settings than in HICs [28],[29].

In one project in Nigeria, village health workers were specifically trained in the recognition of severe mental illness. These workers, who were based in primary care clinics and who had responsibility for case finding in nearby villages, increased the referral rates for cases of schizophrenia [30]. In another program in rural India, community-based rehabilitation workers who were trained to recognize schizophrenia identified and enrolled most of the untreated patients into the program [31],[32].

### Pharmacotherapy

Psychotic conditions can be treated with first-generation antipsychotics (FGAs) such as haloperidol and chlorpromazine or with second-generation antipsychotics (SGAs) such as clozapine and olanzapine. Antipsychotics are generally high-affinity antagonists of dopamine D2 receptors. In LMICs, the usual drug treatment for schizophrenia is an FGA. Evidence from trials undertaken in both HICs [33]–[35] and in China, an LMIC [36], indicates that FGAs are as effective as SGAs for the treatment of the first episode of schizophrenia (Table 1). Meta-analyses that considered evidence collected in HICs indicate that oral haloperidol and chlorpromazine are more effective than placebo [37]–[39], and as effective as the newer antipsychotic compounds in the treatment of the main psychotic symptoms (delusions and auditory hallucinations) of schizophrenia [33],[40],[41]. However, in a naturalistic trial in Brazil olanzapine had advantages over FGAs in terms of improving negative symptoms and quality of life and reducing the incidence of tardive dyskinesia [42],[43]. Finally, a recent meta-analysis of evidence collected in HICs showed that four SGAs (amisulpride, clozapine, olanzapine, and risperidone) were superior to FGAs [44].

**Table 1:** The evidence in support of schizophrenia treatment.

Schizophrenia Treatment	Evidence from HICs	Evidence from LMICs
**Recognition of schizophrenia**	Meta-analysis of early intervention for psychosis [21]	Two longitudinal studies in rural India [31],[32]
**Antipsychotic pharmacotherapy: first episode**	A meta-analysis and two RCTs comparing FGAs and SGAs for the treatment of first psychotic episodes [33]–[35]	RCT from China comparing the FGA haloperidol with the SGA olanzapine for first episode [36]
**Antipsychotic pharmacotherapy**	Meta-analyses of treatment with haloperidol and chlorpromazine [37]–[39]	Naturalistic trial from Brazil comparing olanzapine with FGAs in terms of effects on negative symptoms, quality of life, and the incidence of tardive dyskinesia [42],[43]
	Meta-analyses of treatment with SGAs [39]–[41]	—
	Meta-analysis comparing SGAs with FGAs [44]	—
**Rapid tranquilization**	Meta-analysis of haloperidol plus promethazine for the control of psychosis-induced aggression [45]	Pragmatic RCT in psychiatric emergency settings in India of intra-muscular olanzapine versus intramuscular haloperidol plus promethazine for rapid tranquillization [46]
	—	Pragmatic RCT of intramuscular haloperidol versus intramuscular haloperidol plus promethazine in Brazil [47]
**Long-acting antipsychotics**	Meta-review of depot antipsychotic drugs [48]	—
	Meta-analysis of depot fluphenazine decanoate [49]	—
**Psychosocial family interventions**	Meta-analyses of family psychosocial interventions [52],[53]	Study of a mutual support group for families in Hong Kong [54],[55]
**Psycho-education**	Meta-analysis of psycho-education [56]	RCT of psycho-education in rural China (Xinjin Country, Chengdu) [57]
	—	RCT of family education delivered by nurses in Beijing [58]
	—	RCT of group psycho-education delivered by paramedical staff in Nigeria [59]
**Adherence therapy**	—	RCT of the efficacy of adherence therapy delivered by nurses in Chiang Mai, Thailand [60]
**Community based rehabilitation (CBR)**	—	Two longitudinal studies of CBR in rural India [31],[32]
**Cognitive behavioral therapy (CBT)**	Meta-analysis of the effect of CBT on positive symptoms [61]	—
	RCTs of the effect of CBT on persistent psychotic symptoms [62]–[65]	—
**Promoting recovery-supportive employment**	Meta-analysis of vocational rehabilitation [67]	—
**Wellness training**	RCT of wellness training, health promotion for severely mentally ill adults [66]	—
**Assertive community treatment (ACT)**	Meta-analysis of the effects of ACT on time spent in hospital and ability to live independently [68]	—

RCT, randomised controlled trial.

For agitated patients, evidence collected in HICs [45] and LMICs [46],[47] indicates that the combination of haloperidol and promethazine can effectively control disturbed behavior.

Some patients do not stabilise even after prolonged treatment with an FGA. Sometimes, the use of another antipsychotic may be effective but it is important to explore other causes of nonresponse to treatment, such as nonadherence with the medication regimen, social stressors, or concurrent use of alcohol and/or other drugs before changing a patient's drug regimen. Evidence from meta-analyses of trials undertaken in HICs suggests that a long-acting injectable FGA preparation (for example, depot fluphenazine decanoate or fluphenazine enanthate) can be effective in the case of noncompliance with an oral FGA regime [48],[49].

### Family Education and Psychosocial Interventions

The comprehensive management of schizophrenia includes psychosocial interventions as well as the use of medication. Early findings that a negative family environment (“expressed emotion” that includes high levels of criticism, hostility, or overinvolvement) was related to a poorer prognosis of schizophrenia [50] led to the development of interventions designed to improve the family environment [51]. Several of these strategies have now been shown to enhance functioning in areas such as independent living, relationships, and work. Family interventions targeted at people in close contact with the patient decreased the level of negative expressed emotions, improved compliance with treatment, and averted relapse and hospital admission in two HIC-based meta-analyses [52],[53] and in a Hong Kong-based study that involved a mutual support group delivered by a trained psychiatric nurse [54],[55].

Simpler and less expensive brief psycho-educational interventions that increase knowledge, insight, and management of the illness have also been tested in both HICs and LMICs [56]–[58]. Patients and family members are provided with support, information about medication and the illness, and management strategies, either in small groups or on a one-to-one basis. A meta-analysis of studies undertaken in HICs [56] and two trials undertaken in China [57],[58] show that approaches of this type can improve compliance, decrease relapse, and decrease readmission rates. In addition, compliance with scheduled appointments improved in a study in which four sessions of group psycho-education were given to discharged patients in Nigeria by paramedical staff [59].

Two additional psychosocial approaches have been tested in LMICs. In a randomized controlled trial in Chiang Mai, Thailand, eight sessions of adherence therapy (a behavioral approach designed to enhance compliance with antipsychotic medication) improved psychotic symptoms in patients after 9 wk follow-up [60]. In two studies in rural India, a community-based rehabilitation model in which mental-health workers helped families and clients to recognise symptoms of the disease improved treatment compliance, social inclusion, and social functioning [31],[32]. Additional resources for psychosocial intervention packages are provided in Table S2.

The efficacy of several other psychosocial interventions has been tested only in HICs (Table 1). These include the use of individual cognitive behavioral therapy to decrease positive and persistent symptoms [61]–[64], and suicidal ideation [65], the use of wellness training as health promotion [66], vocational rehabilitation [67], and assertive community care (ACT), a strategy designed to decrease time spent in hospital and to promote living independence [68].

## Delivery of Effective Interventions

The World Health Organization (WHO) ATLAS [69] clearly shows the lack of adequate mental health services in many parts of the world, especially in rural areas in LMICs. The best way to tackle the treatment gap in LMICs is to take advantage of the existing primary health care facilities [70] and to make efficient use of the scarce specialised personnel to train nonspecialist health professionals to identify and manage psychiatric disorders [71],[72]. In India, for example, the training of community-level workers has been shown to be an effective way to improve the detection of schizophrenia and other major mental disorders and to improve their treatment or referral to the appropriate centres of care [31],[32],[73]. To improve access to treatment, providers of mental-health services must make deliberate efforts to raise awareness among communities [74] about the treatability of mental illness and about how to access services. They must also endeavour to reduce the stigma and discrimination associated with mental illness. A recent study suggests that the most effective interventions to achieve this aim are direct social contact with people with mental illness at the individual level and the promotion of social marketing (advertising and promotional methods designed to achieve a social good rather than sales of a commodity) at the population level [75].

In Table 2 and the rest of this section, we describe a series of steps or strategies that might be used to deliver care for schizophrenia in LMICs.

**Table 2:** Delivering schizophrenia treatments.

Step	How	By Whom	In What Settings
**Increasing demand for the package**	Improve mental health literacy in the community [74]	Community-based service staff; community volunteers/extension workers linked to primary health care services, in collaboration with governmental and relevant NGOs	Community
	Key informant identification of cases, direct social contact with people with mental illness at the individual level [32],[75]	—	—
	Community education about causes, treatability, and how to access services [75]	—	—
	Social marketing at the population level [75]	—	—
**Increasing access to the package**	Use local primary health care services and/or general hospitals to deliver the package [70]–[72]	Service providers, governments, insurance providers and users	Community
	Increase acceptability of service by involving service users and community members, establish self-help groups [32]	—	—
**Improving recognition of the disorder**	Train local/traditional practitioners to recognize and refer (key informants)	Local specialists in government/NGO, using international guidelines	Primary care
	Train and supervise community health workers to deliver psycho-education [32],[54],[59],[60]	—	—
	Establish practical protocols/operationalised checklists to improve diagnostic reliability	—	—
**Initiation of evidence based treatments**	Train general physicians and primary health care workers to use antipsychotics [101]–[103]	Local specialists using training materials for medical and psychosocial interventions	Primary care
	Train general physicians and primary health care workers to identify the main side effects of medication (Parkinsonism, akathisia, dystonia, and tardive dyskinesa) [101]–[103]	—	—
	Train and supervise primary health care workers to deliver psycho-educational intervention [54],[57],[58]	—	—
**Managing serious or complex cases**	Train general physicians and primary health care workers to deal with agitation and dangerous behaviour [46],[47]	Training and supervision by local specialists in government/NGO	Primary care
	Train general physicians and primary health care workers to recognize and treat refractory patients with clozapine [39],[101]	—	—
**Achieving optimal outcomes**	Train general physicians and primary health care workers to maintain follow-up with physical routine examinations, assessment of compliance [59],[60], and screen for suicidal thoughts [87]–[89]	Training and supervision by local specialists in government/NGO	Primary care
**Addressing impacts on other health/social outcomes**	Occupation/livelihoods support through access to capital, skills training, inclusive education [32]	User and carer organizations, NGOs, government and alliances, criminal justice system, and interested stakeholders and community members	Community
	Human rights protection, prisoners' health care, access to justice in cases of abuse	—	—
	Advocacy for appropriate, rights-based legislation, and social protection [32]	—	—

NGO, nongovernmental organisation.

### Interventions to Increase Demand for Care Packages

One important aspect of delivering schizophrenia care in LMICs is to ensure that the strategy adopted is tailored to local circumstances. Several trials in LMICs have shown the feasibility of training primary health care workers to undertake psycho-educational activities [54],[55],[57]–[60] that are driven by the local culture and needs. However, because different explanatory models determine help-seeking patterns, people in remote rural areas may find it easier to go to traditional healers and religious centres for help than to a health centre [76],[77]. Furthermore, in many LMICs more than 90% of people with schizophrenia live with their families who manage most aspects of patients' lives. Thus, efforts to increase demand for care packages in such countries should be primarily targeted at families. Similarly, issues such as jobs and employment are best dealt with by a discussion between patients, families, and potential employers in many LMICs [78]. Finally, societies in many LMICs do not believe that overinvolvement (a behaviour marked by an exaggerated emotional response, excessive self-sacrifice, and overprotectiveness) with younger or ill family members is necessarily harmful. Thus, in care packages for LMICs that include aspects of the expressed emotion theory (the idea that negative emotions expressed by family members can be harmful to the person with schizophrenia) [51], baseline measures of expressed emotion must be modified to allow for overinvolvement being routinely higher in LMICs than in Western cultures [79] if demand for the package is to be optimised.

### Interventions to Improve Access to the Package

One assumption behind establishing packages of care for use in LMIC settings is that there is clinical validity in the concept of schizophrenia across diverse populations [80]. Where nonspecialists prescribe medication, it has been argued that treatment of symptoms is more appropriate than arriving at a diagnosis [81]. In the Tanzanian national programme, for example, “acute psychosis” and “chronic psychosis” form the basis for treatment rather than “schizophrenia” [81]. In this type of delivery model, treatment is initiated in the acute phase and all new cases are reviewed within a fixed period of time (for example, a month) if the front-line worker is not a specialist. This type of delivery model reinforces the importance of a system for supervision and referral but also suggests that health workers in LMICs should be trained to deal with transient psychotic disorders [82],[83] and to identify possession states, which might be better managed by traditional healers [84].

A common problem encountered in the treatment of schizophrenia is poor compliance with treatment. This behaviour can be due to lack of understanding of the importance of ongoing treatment for the maintenance of good health and the prevention of relapse or a lack of willingness on the part of the client to take their drugs because of unpleasant side effects. Some patients may refuse to take their drugs because they do not believe they require treatment at all. In other cases, poverty may prevent patients buying drugs or travelling to a care facility. Thus, services that emphasise ease of access, affordability, and family engagement are necessary to overcome these barriers in LMICs [52],[53],[57]–[60].

### Interventions to Manage Serious or Complex Cases

Around 40%–50% of patients with schizophrenia disorders are known to have made at least one suicide attempt and 10% of them complete suicide. A recent study in a HIC found a lifetime suicide prevalence of 4.5%–5.0% for patients with schizophrenia disorder [85]. Despite the new medications, there is little evidence for decreasing rates of suicide among people with schizophrenia [6]. Other evidence, mostly from HICs, indicates that high rates of suicide are associated with drug/alcohol use, severity of symptoms, positive symptoms, first episode psychosis, being a young male, longer duration of untreated psychosis, younger age at the onset of psychosis, recent discharge from hospital, unemployment, hopelessness, high levels of premorbid functioning, high levels of cognitive functions, absence of supportive environment, poor adherence to treatment, and a family history of suicidal attempts [6],[86]. Mental-health workers in LMICs need to be carefully trained to screen and deal with suicidal thoughts and behaviour among patients with schizophrenia [87]–[89].

### Interventions to Manage Impacts on Social Outcomes

People with schizophrenia are more liable to commit crimes than the general population [90]–[92]. The onset of a psychotic episode in an LMIC is more likely to be linked to episodes of assaultive behaviour or contact with police than in a HIC [93],[94]. Furthermore, violent behaviour is seen more frequently in patients with active delusions and hallucinations where there is comorbidity with substance abuse, and in younger persons [90]. Because actual or threatened violent behaviour is a recognised major stimulus for seeking care, mental-health workers in LMICs need to be trained to manage aggressive behaviour, and prison staff should be able to refer patients whose altered behaviour may be due to mental illness.

## Packages of Care for Schizophrenia in LMICs

Our review suggests that recovery in schizophrenia in LMICs can be achieved by using antipsychotics, along with psychosocial education and/or family interventions. The adjunctive use of psychosocial educational strategies can help to improve knowledge and awareness of the condition, lower stigma, and improve understanding of the role of medicines and the importance of compliance to treatment for prevention of relapse. People with schizophrenia may also need support for housing and employment. Although there are few organizations that advocate and defend human rights in LMICs, they can nevertheless actively lobby for better mental-health services. Models for supporting persons with disabilities that emphasise a broad, empowerment- and rights-based approach such as community-based rehabilitation [31],[32] are also applicable to people with mental illness in LMICs. Table 3 proposes a package of care for schizophrenia in LMICs that incorporates these aspects of care and contrasts this package with one that can be provided in HICs.

**Table 3:** Package of care for schizophrenia.

Low Resourced Setting	High Resourced Setting
Community-based rehabilitation	Assertive community care
Psycho-education	Psycho-education
Support for family groups delivered by nonspecialist health workers	Psychosocial family intervention
FGAs and SGAs wherever available	Choice of antipsychotics (SGAs and FGAs)
	Cognitive behavioral therapy
	Supportive employment
	Wellness training

At present, more than a third of patients in LMICs with chronic schizophrenia remain untreated, even when psychiatric services are available [95]. Although both social and clinical factors seem to contribute to this treatment gap, explanatory models of mental illness held by rural people in many of the countries will need to be explored and addressed if this gap is to be reduced [96]. These explanatory models are often rooted in local cultural and religious beliefs and values, which underscores the need for any community care program to consider these factors while planning interventions. Furthermore, because early detection leads to better outcomes in schizophrenia [15],[16], emphasis should be placed on increasing demand for the package, increasing access to the package, and increasing recognition of the disorder (see Table 2).

A recent study conducted in Chile, Nigeria, and Sri Lanka (LMICs that are representative of South America, Africa, and Southeast Asia, respectively) showed that in these countries FGAs constitute a better value choice than SGAs because they are cheaper and more cost-effective [97]. However, as indicated in Table 3, SGAs should be considered in countries where they have a similar cost to FGAs because of the lower incidence of extrapyramidal side effects with these drugs [43],[44]. Importantly, other studies done in LMICs indicate that the combination of pharmacotherapy and psycho-educational interventions is more cost-effective than the use of drugs alone [97]–[99].

As shown in Table 3, specialist services for particular groups such as cognitive behaviour therapy or assertive outreach services should be reserved for high resource settings [100]. Furthermore, although inpatient beds are a necessary component of a comprehensive package of care—particularly for the treatment of severe, acute episodes of psychosis—these beds should be available in the mental-health wards of general hospitals in LMICs rather than solely in specialist hospitals [100]. Admissions in this environment are generally more locally accessible, more affordable, and less stigmatising. These general hospital psychiatry departments can also provide direct supervision and secondary referral services to local practitioners, whereas tertiary-level specialist hospitals can focus more on training, research, and advocacy for improved national systems for delivery of mental-health care.

In summary, in LMICs general physicians and primary health care workers can be trained to recognise and treat people with psychotic disorders in the community. A package of care that combines low doses of conventional antipsychotics along with brief, simple psycho-educational interventions is recommended as an important strategy to decrease the treatment gap for schizophrenia in LMICs. In our view, the treatment package presented here, if combined with a scale-up of mental-health services in LMICs [72], should provide both a feasible and a cost-effective way to extend treatment to many more patients with schizophrenia who live in LMICs, and should have a major positive impact upon their lives and upon the lives of their families.

## Supporting Information

Table S1 Mental health resources in LMICs.
(0.17 MB DOC)

Table S2 Family education resources and training materials for health workers.
(0.04 MB DOC)

## Abbreviations

FGA: first-generation antipsychotic
HIC: high-income countries
LMIC: low- and middle-income countries
SGA: second-generation antipsychotic
