# Supplementary material for: Packages of Care for Schizophrenia in Low- and Middle-Income Countries
Source: PLoS Med. 2009 Oct 20;6(10):e1000165. doi: 10.1371/journal.pmed.1000165 (PMC2758997; doi:10.1371/journal.pmed.1000165)
Supplement: Table S1 — Mental health resources in LMICs. (0.17 MB DOC) [file pmed.1000165.s001.doc]

**Table S2 - Family education resources and training materials for health workers**

**Family education Resources**

In English unless otherwise stated

American Psychiatric Association (USA)

Public information campaign including website for accessible information

[www.healthminds.org](http://www.healthminds.org/)

Hong Fook Mental Health Association (Canada)

Information in English and Chinese

[http://www.hongfook.ca](http://www.hongfook.ca/)

National Institute of Mental Health (USA)

Easy to understand information, some also in Spanish

<http://www.nimh.nih.gov/health/publications/easy-to-read.shtml>

Royal College of Psychiatrists (UK)

Information leaflets for users and carers (some leaflets in up to 14 languages)

<http://www.rcpsych.ac.uk/mentalhealthinfoforall.aspx>

World Federation for Mental Health

Wide range of information, much of it aimed at users and carers.

[http://www.wfmh.org](http://www.wfmh.org/)

World Health Organisation

Comprehensive information pack for families of people with schizophrenia

<http://whqlibdoc.who.int/HQ/1992/WHO_MNH_MND_92.8.pdf>

**Training materials for health workers**

Amaudo UK

*Mental Health and Human Rights – Learning Pack for Village Health Workers*

The Amaudo Training Unit, Amaudo UK

[http://www.amaudouk.org](http://www.amaudouk.org/)

Annan, J., Castelli, L., Devreux, A., Haworth, H., Kerins, M. (2000). *AVSI Training Manual for Community Volunteer Counselors*. AVSI & UNICEF: Kampala, Uganda.

<http://www.avsi.org/documenti/CaseStudyUganda.pdf>

The International Federation of Red Cross and Red Crescent Societies *Community-Based Psychological Support: Training Manual.*

IFRC, 2003

<http://www.ifrc.org/what/health/psycholog/manual.asp>

Richards, D., Tradshaw, T. & Mairs, H.

*Helping People with Mental Illness: A Mental Health Training Programme for Community Health Workers*.

University of Manchester, 2003

[http://www.who.int/mental_health/policy/en/Module%20D.pdf](http://www.who.int/mental_health/policy/en/Module D.pdf)

Schizophrenia Research Foundation (SCARF)

*Manual for training of Community Mental Health Workers*

Contact; Dr.R.Thara, Director, SCARF, Chennai, India

[www.scarfindia.org](http://www.scarfindia.org/)

Weel-Baumgarten, E. van, Mynors-Wallis, L., Jané-Llopis, E., & Anderson, P.

*A training manual for prevention of mental illness: managing emotional symptoms and problems in primary care.*

Implementing Mental Health Promotion Action (IMHPA). Nijmegen: Radboud University of Nijmegen, 2005. <http://www.gencat.net/salut/imhpa/Du32/html/en/dir1662/dd11713/imhpa_training_manual.pdf>

World Health Organization

*Human Resources and Training in Mental Health. Mental Health Policy and Service guide Package.*

WHO: Geneva, 2005

<http://www.who.int/mental_health/policy/Training_in_Mental_Health.pdf>

World Health Organization; Nations for Mental health Project.

*Psychiatric Notes for Volunteer Community Workers*.

WHO/Ghana Ministry of Health, 2003.

[http://www.who.int/mental_health/policy/en/Ghana_psychiatric%20notes%20for%20volunteers.pdf](http://www.who.int/mental_health/policy/en/Ghana_psychiatric notes for volunteers.pdf)

World Health Organization (1998) *Mental disorders in primary care. A WHO Educational Package.*

WHO: Geneva, 1998

<http://whqlibdoc.who.int/hq/1998/WHO_MSA_MNHIEAC_98.1.pdf>
