# Supplementary material for: Packages of Care for Schizophrenia in Low- and Middle-Income Countries
Source: PLoS Med. 2009 Oct 20;6(10):e1000165. doi: 10.1371/journal.pmed.1000165 (PMC2758997; doi:10.1371/journal.pmed.1000165)
Supplement: Table S2 — Family education resources and training materials for health workers. (0.04 MB DOC) [file pmed.1000165.s002.doc]

**Table S1. Mental health resources in low and middle income countries**

|  | World  Bank  classification | Language (a) | Population  (million)  (b) | Number of psychiatrists/ 100,000 inhabitants (a) | Number of general physicians/  10,000 inhabitants (c) | Number of psychiatric beds/  10,000 (a) | Expenditure with health as % GDP(a) | Total Expenditure in health (per capita)U$ (a) | Public expenditure in health (per capita)U$ (a) | Expenditure in mental health as % global health  budget (a) |
| --- | --- | --- | --- | --- | --- | --- | --- | --- | --- | --- |
| Africa - North and Western |  |  |  |  |  |  |  |  |  |  |
| Egypt, Arab Rep. | LMIC | Arabic | 81,713 | 0.9 | 24.0 | 1.3 | 3.9 | 153 | 75 | 9.0 |
| Libya | UMIC | Arabic, Italian and English | 6,173 | 0.18 | 13.0 | 1.0 | 2.9 | 239 | 134 | NA |
| Mauritania | LIC | Arabic, Fula, Soninke, Wolof and French | 3,364 | 0.08 | 1.0 | 0.2 | 3.6 | 45 | 33 | 1.0 |
| Morocco | LMIC | Arabic | 34,343 | 0.4 | 5.0 | 0.78 | 5.1 | 199 | 78 | NA |
| Sudan | LIC | Arabic and English | 40,218 | 0.09 | 3.0 | 0.2 | 3.5 | 39 | 7 | NA |
| Africa- Sub-Sahara |  |  |  |  |  |  |  |  |  |  |
| Angola | LIC | Portuguese, Bantu, Ovimbundu, Kimbundu and Kikongo | 12,531 | 0 | <1.0 | 0.13 | 4.4 | 70 | 44 | NA |
| Botswana | LIC | English and Setswana | 1,842 | 0.4 | 4.0 | 1.1 | 6.6 | 381 | 252 | 1.0 |
| Burkina Faso | LIC | French | 15,264 | 0.05 | <1.0 | 0.18 | 3.0 | 27 | 16 | NA |
| Burundi | LIC | French and Swahili | 8,691 | 0.02 | <1.0 | 0.1 | 3.6 | 19 | 11 | NA |
| Cameroon | LIC | French and English | 18,467 | 0.03 | 2.0 | 0.08 | 3.3 | 42 | 16 | 0.1 |
| Cape Verde | LIC | Portuguese and Criuolo | 0,426 | 0.9 | 5.0 | 0.78 | 4.5 | 165 | 138 | NA |
| Central African Republic | LIC | French | 4,444 | 0.03 | <1.0 | 0.07 | 4.5 | 58 | 30 | NA |
| Chad | LIC | French and Arabic | 10,111 | 0.01 | <1.0 | 0.02 | 2.6 | 17 | 13 | NA |
| Comoros | LIC | French, Arabic and Swahili | 0,731 | 0 | 2.0 | 0 | 3.1 | 29 | 17 | NA |
| Congo, Dem. Rep. | LIC | French | 66,514 | 0.04 | 1.0 | 0.17 | 3.5 | 12 | 5 | NA |
| Congo, Rep. | LIC | French | 3,903 | 0.03 | 2.0 | 0.06 | 2.1 | 22 | 14 | NA |
| Côte d'Ivoire | LIC | French, Akan and Mandés | 20,179 | 0.2 | 1.0 | 0.15 | 6.2 | 127 | 20 | 0.1 |
| Djibouti | LIC | Arabic and French | 0,506 | 0 | 2.0 | 0.7 | 7.0 | 90 | 53 | NA |
| Equatorial Guinea | LIC | Spanish, French, Ndowe, Bisio and Annobonés | 0,616 | 0 | 3.0 | 0 | 2.0 | 106 | 64 | NA |
| Eritrea | LIC | Tigrigna, Tigre, Saho, Afar, Bilen, Kunama, Nara, Hidarib and Arabic | 5,502 | 0.03 | <1.0 | 0.64 | 5.7 | 36 | 23 | NA |
| Ethiopia | LIC | Amharic, Oromo and Tigrinya | 82,544 | 0.02 | <1.0 | 0.07 | 3.6 | 14 | 6 | NA |
| Gabon | UMIC | French, Fang, Punu and Nzèbi. | 1,485 | 0.3 | 3.0 | 0.7 | 3.6 | 197 | 94 | 0.3 |
| Gambia, The | LIC | English | 1,735 | 0.08 | 1.0 | 0.78 | 6.4 | 78 | 39 | NA |
| Ghana | LIC | English | 23,282 | 0.08 | 2.0 | 1.03 | 4.7 | 60 | 36 | 0.5 |
| Guinea | LIC | French | 9,806 | 0.04 | 1.0 | 0.05 | 3.5 | 61 | 33 | NA |
| Guinea-Bissau | LIC | Portuguese | 1,503 | 0 | 1.0 | 0 | 5.9 | 37 | 20 | 2.3 |
| Kenya | LIC | English and Swahili | 37,953 | 0.2 | 1.0 | 0.4 | 7.8 | 114 | 24 | 0.01 |
| Lesotho | LIC | English and Sesotho | 2,128 | 0.05 | <1.0 | 0.8 | 5.5 | 101 | 80 | 7.0 |
| Liberia | LIC | English | 3,334 | 0.03 | <1.0 | 0.08 | 4.3 | 127 | 97 | NA |
| Madagascar | LIC | Malagasy and French | 20,042 | 0.08 | 3.0 | 0.17 | 2.0 | 20 | 13 | 0.8 |
| Malawi | LIC | English and Chichewa | 13,931 | 0 | <1.0 | 0.37 | 7.8 | 39 | 14 | 2.0 |
| Mali | LIC | French | 12,324 | 0.06 | <1.0 | 0.2 | 4.3 | 30 | 12 | 0.02 |
| Mauritania | LIC | Arabic, Fula, Soninke, Wolof and French | 3,364 | 0.08 | 1.0 | 0.2 | 3.6 | 45 | 33 | 1.0 |
| Mauritius | UMIC | English, French and Creole Patois | 1,274 | 1.0 | 11.0 | 9.5 | 3.4 | 323 | 192 | 0.3 |
| Mozambique | LIC | Portuguese (official), Emakua, Xichangana and Elomwé. | 21,284 | 0.04 | <1.0 | 0.23 | 5.9 | 47 | 32 | NA |
| Namibia | LMIC | Afrikaans, German and English | 2,088 | 0.2 | 3.0 | 1.5 | 7.0 | 342 | 232 | NA |
| Niger | LIC | French, Kanuri, Arabic, Gourmantche, Toubou and Boudouma | 13,272 | 0.04 | <1.0 | 0.2 | 3.7 | 22 | 9 | NA |
| Nigeria | LIC | English, Hausa, Yoruba, Igbo and Pidgin English | 146,255 | 0.09 | 3.0 | 0.4 | 3.4 | 31 | 7 | NA |
| Rwanda | LIC | Kinyarwanda, French, English and Swahili | 10,186 | 0.03 | <1.0 | 0.2 | 5.5 | 44 | 24 | 1.0 |
| Samoa | LMIC | Samoan and English | 0,217 | 0 | 3.0 | 0.2 | 5.8 | 199 | 164 | NA |
| São Tomé and Principe | LIC | Portuguese | 0,206 | 0 | 5.0 | 2.2 | 2.3 | 22 | 15 | NA |
| Senegal | LIC | French, Wolof and Serer | 12,853 | 0.16 | <1.0 | 0.3 | 4.8 | 63 | 37 | 9.0 |
| Sierra Leone | LIC | English | 6,294 | 0.02 | <1.0 | 0.47 | 4.3 | 26 | 16 | NA |
| Somalia | LIC | Somali, Arabic, English and Italian | 9,558 | 0.06 | <1.0 | 0.39 | 2.6 | 15 | 7 | NA |
| Sudan | LIC | Arabic and English | 40,218 | 0.09 | 3.0 | 0.2 | 3.5 | 39 | 7 | NA |
| Swaziland | LMIC | English and Swazi | 1,128 | 0.1 | 2.0 | 2.0 | 3.3 | 167 | 115 | 0.3 |
| Tanzania, United Rep. | LIC | Swahili and English | 40,213 | 0.04 | <1.0 | 0.7 | 4.3 | 26 | 12 | 7.0 |
| Togo |  | French, Ewé, Mina, Kabyé and Cotocoli | 5,858 | 0.04 | <1.0 | 0.4 | 2.8 | 45 | 22 | 0.2 |
| Zambia | LIC | English | 11,669 | 0.02 | 1.0 | 0.5 | 5.7 | 52 | 27 | NA |
| Zimbabwe | LIC | English, Ndebele and Shona | 11,350 | 0.1 | 2.0 | 1.2 | 6.2 | 142 | 64 | NA |
| Asia - South Central |  |  |  |  |  |  |  |  |  |  |
| Afghanistan | LIC | Pashtu, Dari Persian and Turkic | 32,738 | 0.036 | 2.0 | 0.055 | 5.2 | 34 | 18 | NA |
| Bangladesh | LIC | Bangla | 153,546 | 0.05 | 3.0 | 0.065 | 3.5 | 58 | 26 | 0.5 |
| Bhutan | LIC | Chhokey, Ngalopkha Sharchopkha and Nepali | 0.682 | 0.3 | <1.0 | 0 | 3.9 | 64 | 58 | 0.17 |
| India | LIC | Hindi, English | 1,147,995 | 0.2 | 6.0 | 0.25 | 5.1 | 80 | 14 | 2.0 |
| Maldives | LMIC | Dhivehi | 0,385 | 0.36 | 9.0 | NA | 6.7 | 263 | 220 | NA |
| Nepal | LIC | Nepali | 29,519 | 0.12 | 2.0 | 0.08 | 5.2 | 63 | 19 | 0.08 |
| Pakistan | LIC | Punjabi, Sindhi, Siraiki, Pashtu and Urdu | 172,800 | 0.2 | 8.0 | 0.24 | 3.9 | 85 | 21 | 0.4 |
| Sri Lanka | LMIC | Sinhala | 21,128 | 0.2 | 6.0 | 1.8 | 3.6 | 122 | 60 | 1.6 |
| Asia - Southeast |  |  |  |  |  |  |  |  |  |  |
| Burma (Myanmar) | LIC | Burmese | 47,758 | 0.2 | 4.0 | 0.55 | 2.1 | 26 | 5 | 1.3 |
| Cambodia | LIC | Khmer | 14,241 | 0.16 | 2.0 | 0 | 11.8 | 184 | 27 | NA |
| Indonesia | LMIC | Bahasa Indonesia | 237,512 | 0.21 | 1.0 | 0.4 | 2.4 | 77 | 19 | 1.0 |
| Lao PDR | LIC | Lao | 6,677 | 0.03 | 4.0 | 0.07 | 3.1 | 51 | 29 | NA |
| Malaysia | UMIC | Malay, Chinese, Tamil and English. | 25,274 | 0.06 | 7.0 | 2.7 | 3.8 | 345 | 185 | 1.5 |
| Philippines | LMIC | Pilipino and English | 96,061 | 0.4 | 12.0 | 0.9 | 3.3 | 169 | 77 | 0.02 |
| Thailand | LMIC | Thai, Chinese and English | 65,493 | 0.6 | 4.0 | 1.4 | 3.7 | 254 | 145 | 2.5 |
| Timor-East | LIC | Tetum and Portuguese | 1,108 | NA | 1.0 | NA | 9.8 | NA |  | NA |
| Vietnam | LIC | Vietnamese | 86,116 | 0.32 | 6.0 | 0.63 | 5.1 | 134 | 38 | NA |
| Asia - Western |  |  |  |  |  |  |  |  |  |  |
| Iraq | LMIC | Arabic and Kurdish. | 28,221 | 0.7 | 7.0 | 0.63 | 3.2 | 97 | 31 | NA |
| Jordan | LMIC | Arabic | 6,198 | 1.0 | 24.0 | 1.57 | 9.5 | 412 | 194 | NA |
| Syrian Arab Republic | LMIC | Arabic, French and English | 19,747 | 0.5 | 5.0 | 0.8 | 5.4 | 427 | 188 | NA |
| Turkey | LMIC | Turkish | 71,892 | 1.0 | 16.0 | 1.3 | 5.0 | 295 | 209 | NA |
| Yemen, Rep. | LIC | Arabic | 23,013 | 0.5 | 3.0 | 1.85 | 4.5 | 69 | 24 | NA |
| Latin America and Caribe |  |  |  |  |  |  |  |  |  |  |
| Bolivia | LMIC | Spanish, Quechua and Aymara | 9,247 | 0.9 | 12.0 | 0.79 | 5.3 | 125 | 85 | 0.2 |
| Dominica | UMIC | English | 0,072 | NA | 5.0 | NA | 6.0 | 312 | 222 | 2.9 |
| El Salvador | LMIC | Spanish. | 7,066 | 0.5 | 12.0 | 0.65 | 8.0 | 376 | 175 | NA |
| Grenada | UMIC | English | 0,090 | 1.0 | 10.0 | 10.8 | 5.3 | 445 | 320 | 10 |
| Guatemala | LMIC | Spanish and Indian (Maya | 13,002 | 0.54 | 9.0 | 0.35 | 4.8 | 199 | 96 | 0.9 |
| Guyana | LMIC | English | 0,770 | 0.2 | 5.0 | 3 | 5.3 | 215 | 171 | NA |
| Haiti | LIC | French and Creole | 8,924 | NA | 3.0 | NA | 5.0 | 56 | 30 | NA |
| Honduras | LMIC | Spanish | 7,639 | 0.76 | 6.0 | 0.6 | 6.1 | 153 | 81 | 2.3 |
| Nicaragua | LIC | Spanish | 5,785 | 0.64 | 4.0 | 0.34 | 7.8 | 158 | 77 | 1.0 |
| St. Kitts and Nevis | UMIC | English | 0,039 | NA | 11.0 | NA | 4.8 | 576 | 382 | NA |
| St. Vincent and the Grenadines | UMIC | English and French | 0,118 | 0.9 | 8.0 | 10.6 | 6.1 | 358 | 227 | 4.6 |
| Trinidad and Tobago | UMIC | English | 1,231 | 1.0 | 8.0 | 10.29 | 2.5 | 388 | 168 | NA |
| Micronesia and Melanesia |  |  |  |  |  |  |  |  |  |  |
| Fiji | LMIC | Fijian, Hindi, English and Rotuman | 0,931 | 0.25 | 5.0 | 2.34 | 4.0 | 224 | 150 | 1.7 |
| Kiribati | LMIC | English | 0,110 | 1.0 | 2.0 | 7.3 | 8.6 | 143 | 141 | 1.6 |
| Marshall Islands | LMIC | Marshallese and English | 0,063 | 0 | 5.0 | 0 | 9.8 | 343 | 222 | 0.4 |
| Micronesia, Fed. Sts | LMIC | English and Micronesian languages | 0,107 | 0 | 6.0 | 0.7 | 7.8 | 319 | 230 | 7.3 |
| Nauru | HIC | Nauruan | 0,013 | 0 | 8.0 | 0 | 7.5 | 1015 | 900 | NA |
| Papua New Guinea | LIC | English, Tok Pisin and Hiri Motu | 5,931 | 0.09 | <1.0 | 0.24 | 4.4 | 144 | 128 | 0.7 |
| Solomon Islands | LIC | English and Solomon Pijin | 0,581 | 0 | 1.0 | 0.26 | 5.0 | 133 | 124 | 1.4 |
| Vanuatu | LMIC | Bislama, English and French | 0,215 | 0 | 1.0 | 0.1 | 3.8 | 107 | 63 | NA |
| Polynesia |  |  |  |  |  |  |  |  |  |  |
| Cook Islands | HIC | English and Maori | 0,012 | 0 | 12.0 | 0 | 4.7 | 598 | 404 | NA |
| Niue | HIC | Niuean and English | 0,144 | 0 | 20.0 | 0 | 7.7 | 1041 | 1010 | NA |
| Tonga | LMIC | Tongan and English | 0,119 | 1.0 | 3.0 | 2.6 | 5.5 | 223 | 138 | 0.5 |
| Tuvalu | HIC | Tuvaluan and English | 0,012 | 0 | 9.0 | 2.0 | 5.4 | 673 | 359 | NA |

1. WHO, Atlas Project 2005; (b) CIA, The World Factbook (Population – July , 2008); (c) WHO, Statistical Information System (WHOSIS)

Note: The total world population (Source- World Population Prospects: The 2006 Revision population) is about is 6, 906, 558 people and it is estimated 49,797 millions of people suffering from schizophrenia. LAMIC account for 84% of people suffering from schizophrenia (the population of LAMIC is about 5,792,967 and it is estimated 41,709 millions of people with schizophrenia): 16% in African countries and almost 70% in Asian countries. However, it is estimated that 23 millions of people with schizophrenia - almost half of people suffering from schizophrenia in the world - live in 93 LAMIC (table S1) with less than 1 psychiatrist/100,000 inhabitant (population = 2,516,450): 27.% in African countries and 70.5% in Asia countries.
